# Transactions of American Medical Association

**Published:** 1855-03

**Authors:** 


### BIBLIOGRAPHICAL NOTICES.

**THE TRANSACTIONS OF THE AMERICAN MEDICAL ASSOCIATION, Instituted 1847, Vol. VII.** New York: Charles B. Norton, 1854—8vo. pp. 668.

The publication of the Transactions was, at the last meeting at St. Louis, in May, transferred from Philadelphia, to New York; and the committee who have issued the present volume, actuated probably by a laudable zeal, have completed their work at a very early day, and seem also to have done it very faithfully. We certainly shall vote for the present muslin binding, instead of the French-ish pasteboard package, which we have heretofore put up with. The work is furnised with two uncolored engravings and a few maps, illustrating the Reports of epidemics.

First, in the volume, are the proceedings of the seventh Annual

Meeting, of the Association, and the Address of the Vice-President, Dr. USHER PARSONS, of Providence.

*Report of the Committee on Medical Education*, prepared by the Chairman, Prof. CABELL, of the University of Virginia, who has been a prominent advocate of reform in this matter. This report is a clear and able document, and although we might possibly demur at some of the points, still it will well pay attention.

*Report of Committee on the Epidemics of Kentucky, and Tennessee.* By W. L. SUTTON, M. D., Georgetown Ky.—Comprises over fifty pages, and embraces many points of interest.

*On Erysipelas.* By R. S. HOLMES, M. D., of St. Louis, Mo. —While this report has many creditable points, we think the least satisfactory portion is that relating to the treatment. We cannot but wish the author had paid more attention to this part of the subject.

*On the Medical and Toxicological Properties of the Cryptogamic Plants of the United States.* By F. B. PORCHER, M. D., of Charleston S. C. A very elaborate paper, comprising some 125 pages and replete with valuable details respecting the localities, varieties and properties, both medicinal and poisonous of all the cryptogamic plants in this country.

*Report on the Epidemics of Ohio, Indiana and Michigan*, for the years 1852 and '53. By GEORGE MENDENHALL, M. D., aided by numerous assistants ; one hundred and thirty pages, giving a clear and apparently full history of the subjects in question.

*Report on the Epidemics of Louisiana, Mississippi, Arkansas, and Texas, in the year* 1853. By E. D. FENNER, M. D. of New Orleans La. Contains over 130 pages, almost exclusively devoted to a very valuable history of the Yellow Fever, comprising a full consideration of the various questions of interest which arise in connection with this fearful disease.

*Prize Essay: On a New Method of Treating Ununited Fractures and Certain Deformities.* By DAVID BRAINARD, M. D., Prof. of Surg. in Rush Medical College. The object of this essay is stated to be

1st. To establish, by experiment, the principles upon which the treatment of ununited fractures should be conducted, and to show that these principles apply to the human subject.

2nd. To propose a new method of treatment of certain deformities which result from true anchylosis, union of fractures in an angular position, rachitic curvature, &c. The author does not pretend to have been the first to turn his attention to similar treatment; but he does claim "to have demonstrated the principles upon which it reposes, to have laid down the rules by which it should be performed and to have contrived an instrument by which it could be effected with safety and facility."

The author states, as the result of numerous experiments, that,

1. Foreign bodies, of every kind, placed or allowed to remain in contact with any part of a bone, in a manner to keep up suppuration, produce absorption of it, and have no tendency whatever to give rise to the production of callus. The use of setons, pegs, wires, and foreign bodies of every description, as a means of promoting the formation of callus, is a practice not founded on correct principles, and is often dangerous. The seton is more properly employed for the purpose of dividing a bone, or keeping up a false articulation, than for uniting it.

2. Sequestra and foreign bodies, imbedded in bone, may be brought to the surface when, by perforation or otherwise, instruments or ligatures can be so attached to them as to draw them permanently, with but a moderate degree of force, in that direction. As soon as they press against the living bone, they cause its absorption in the direction toward which they are drawn.

3. That power of absorption of bony tissue attributed to the periosteum and the medullary membrane, exists also in the substance of the bone itself, as is proved by the insertion of pegs into perforations of bone, absorption taking place around them, not only at the surfaces, but at all the intervening points.

The author next enquires as to "the effects of foreign bodies on the formation of callus," and concludes that "foreign bodies produce absorption of callus, when placed in contact with it," and medicinal solutions, placed in contact with bones, were found to produce similar effects.

Chapter five, treats of "wounds of bone and the circumstances in which they most readily give rise to callus, or to union without it." The views of the author, respecting the treatment of ununited fractures, are indicated in the following paragraph:

"The careful examination of a false joint, serves to show that something more than an irritation of the soft part is required to form callus upon surfaces already incrusted with cartilage, and surrounded by ligamentous tissue.

The object to be desired in these cases is, as it appears to me, the production, by subcutaneous wound of fresh osseous surfaces opposite to and in contact with each other, with such division of the soft parts as will excite a moderate inflammation, without suppuration, around the false joint; and a wound of the bones, of considerable depth and size, capable of producing that vascularity and softening always noticed in fractured extremities of bones, before union takes place. These fresh surfaces, in some cases, require to be numerous, and equal in the aggregate to those produced by resection; and the operation by which they are made should be repeated from time to time. Hence, it must be an operation which does not expose to violent inflammation, or give great pain. It should, moreover, be one capable of being employed in connection with other methods, recognized as useful or necessary to preserve immobility.

For the accomplishment of these purposes, Prof. B. has invented an instrument for producing subcutaneous puncture of the fractured extremities, capable of penetrating the hardest bone or ivory in any direction, with facility. The method of using it is fully explained in the treatise, and illustrated with two useful engravings. To more fully elucidate the practice alluded to, seven cases are narrated, in which this method was successfully employed. Three of these occurred in the practice of Prof. Brainard, two are reported by Prof. Mussey, of Cincinnati, and two by Dr. Jewett, of New York State. Much credit is due the distinguished author of this Prize Essay for the ingenuity of his investigations, and the practical tact with which he has applied the principles upon which this beautiful practice is based.

*Report on the Norwalk Disaster*, with biographical sketches of those Members who lost their lives on that occasion. This brief and touching narrative calls up again the vivid emotions excited in the breast of every one, by that fearful and criminal wreck of human life. Of the forty-four persons suddenly killed, seven were eminent physicians of New England.

*Remarks.* By Dr. LINTON, on Bilious and Yellow Fevers, follow, and the volume concludes with Reports of Committee of Publication and of Treasurer, and a catalogue of Officers and permanent Members.

